# Will intravenous paracetamol crush fentanyl in patients undergoing PCI for STEMI?

**DOI:** 10.1007/s12471-019-1262-1

**Published:** 2019-03-07

**Authors:** J. M. ten Berg, D. M. F. Claassens

**Affiliations:** Department of Cardiology, St. Antonius ziekenhuis, Nieuwegein, The Netherlands

Pain relief is important in patients experiencing ST-elevation myocardial infarction (STEMI). Not only because pain management improves patient comfort, but also because pain is associated with activation of the sympathetic nervous system, causing vasoconstriction and increased workload of the heart [1]. Therefore, the 2012 STEMI guideline of the European Society of Cardiology (ESC STEMI) recommended administering titrated intravenous opioids (class I, level of evidence (LoE) C) [2]. Since then, multiple studies have shown that morphine causes slower uptake and delayed onset of action of oral antiplatelet agents leading to high platelet reactivity [3, 4]. Furthermore, it could cause nausea and vomiting, leading to the absence of any form of oral P2Y12 inhibition [1]. Therefore, the recommendation was downgraded to class IIa, LoE C, in the 2017 ESC STEMI guideline.

However, whether this higher platelet reactivity actually results in more myocardial injury and increased adverse events (e. g. stent thrombosis) remains unclear as there are no prospective trials on the subject. There are, however, multiple retrospective studies. A study by Gwag et al. included 332 patients with STEMI between 2008 and 2014 who underwent cardiac magnetic resonance imaging. They used propensity score matching to match 90 patients with morphine and 90 patients without morphine, and found no difference in the myocardial salvage index [5]. Multiple other studies investigating clinical outcomes after morphine use in STEMI patients also found no differences between patients with morphine and patients without [6, 7]. We do know that it takes several hours to reach sufficient inhibitory effect on platelets, even with the use of ticagrelor [8]. An explanation for the discrepancy of this delayed onset of action and the absence of a clinical effect, might be that the administration of other intravenous antiplatelet and antithrombotic agents, such as aspirin, heparin and glycoprotein IIb/IIIa inhibitors, before, during and after primary percutaneous coronary intervention (PCI) might mitigate the extra delayed onset of action after morphine use. Another explanation for the discrepancy might be that the studies were simply underpowered to prove any differences between groups. A study which was not underpowered, was the Crusade (Can Rapid Risk Stratification of Unstable Angina Patients Suppress Adverse Outcomes with Early Implementation of the ACC/AHA Guidelines) study, which included 57,039 non-STEMI patients between 2001 and 2003 [9]. In this retrospective analysis, morphine use was associated with an increased risk of in-hospital death and myocardial infarction. However, these patients did not routinely undergo primary PCI and only a minority of the patients received a P2Y12 inhibitor (40%). It is therefore questionable whether these results can be extrapolated to the current STEMI population.

As long as the effects of morphine on clinical outcomes are unsure, the search for other solutions should continue. The ON-TIME 3 randomised controlled trial, which is presented in the current issue of the Netherlands Heart Journal by Tavenier et al. will investigate patients receiving intravenous paracetamol on top of crushed ticagrelor compared with patients receiving intravenous fentanyl on top of crushed ticagrelor. This could be of great additional value to help determine the optimal treatment strategy of STEMI patients and improve our knowledge about the pharmacodynamics of ticagrelor [10]. Especially interesting, in our opinion, will be the result of crushed ticagrelor on timing of complete platelet inhibition. Recently, crushed ticagrelor has been suggested as a better option than standard ticagrelor tablets in several smaller STEMI trials, demonstrating a significantly faster reduction in platelet reactivity as early as 30 minutes after administration [11, 12]. Intravenous platelet inhibitors, such as the P2Y12 inhibitor cangrelor, and the glycoprotein IIb/IIIa inhibitors abciximab and tirofiban are associated with an even earlier reduction in platelet reactivity [13, 14]. However, due to the high costs of these agents as compared with ticagrelor, and a lack of trials comparing the two on clinical endpoints, this is not routinely indicated [15]. The use of crushed ticagrelor could mean sufficient platelet inhibition would still be achieved during primary PCI. It will be interesting to see if the results of the smaller trials can be replicated. Furthermore, it will be interesting to see if 1000 mg of intravenously administered paracetamol, which has an infusion time of 15 minutes, is a strong enough analgesic and can achieve the analgesic effect within adequate time to overcome the pain and discomfort caused by myocardial infarction when compared with intravenous fentanyl, which has an infusion time of just 30 seconds. Prior research on this subject has not been published and if paracetamol is proven to have a sufficient analgesic effect in most cases, this could have a major impact on the pre-hospital treatment of STEMI patients worldwide. That is why we are looking forward to the results of the ON-TIME 3 trial by Tavenier et al.
